# Supplementary material for: Gene regulatory networks reveal sex difference in lung adenocarcinoma
Source: Biol Sex Differ. 2024 Aug 6;15:62. doi: 10.1186/s13293-024-00634-y (PMC11302009; doi:10.1186/s13293-024-00634-y)
Supplement: Supplementary file 1 — Supplementary Material 1 A. Designing Sex-specific Transcription Factor-Gene Motif Prior. B. Designing Protein-protein Interaction Prior. C. Sex Difference in anti PD-1 and anti PDL-1 Inhibitors in Non-small Cell Lung Cancer. Figure D.6: Defining biological sex based on sex chromosome complement. Scatterplot of first two principal components of Y chromosome gene expression in GTEx (top left), TCGA (top right), LGRC (bottom left) and GSE68465 (bottom right). Figure D.1: Sex difference in LGRC control lung samples within nonsmokers and smokers. Normalized enrichment scores (NES) from GSEA using KEGG pathways are shown for all pathways that have significant (adjusted p-value < 0.05) sex difference among either nonsmokers or smokers in LGRC. Pathways with higher targeting in male are marked blue and pathways with higher targeting in female are marked red. Green boxes highlight pathways associated with cell proliferation and brown boxes highlight pathways associated with environmental carcinogen metabolism. Figure D.2: Sex difference in tumor samples from the validation data GSE68465 within nonsmokers and smokers. Normalized enrichment scores (NES) from GSEA using KEGG pathways are shown for all pathways that have significant (adjusted p-value < 0.05) sex difference among either nonsmokers or smokers (in TCGA). Pathways with higher targeting in male are marked blue and pathways with higher targeting in female are marked red. Green boxes highlight pathways associated with cell proliferation and purple boxes highlight pathways associated with immune response. Figure D.5: Sex difference in immune and stromal cell composition in GTEx samples: nonsmokers (left) and smokers (right). Cell compositions are computed using “xcell”, which derives cell composition proportion of 36 immune and stromal, along with three composite scores: immune score, stroma score and microenvironment score. The bubbleplot shows only those cells that are significantly (p-value < 0.05) different in prop [file 13293_2024_634_MOESM1_ESM.docx]

**Gene regulatory Networks Reveal Sex Difference in Lung Adenocarcinoma**

**Supplementary Materials**

Enakshi Saha^1^, Marouen Ben Guebila^1^, Viola Fanfani^1^, Jonas Fischer^1^, Katherine Hoff-Shutta^1,2^, Panagiotis Mandros^1^, Dawn L DeMeo^2,3^, John Quackenbush^1,2,4^, Camila M Lopes-Ramos^1,2,3^

^1^ Department of Biostatistics, Harvard T. H. Chan School of Public Health, Boston, MA 02115, USA

^2^ Channing Division of Network Medicine, Brigham and Women’s Hospital, Boston, MA, USA 02115

^3^ Department of Medicine, Harvard Medical School, Boston, MA 02115, USA

^4^ Department of Data Science, Dana-Farber Cancer Institute, Boston, MA 02115, USA

**Corresponding Author:** Camila M Lopes-Ramos

Email: [nhclr@channing.harvard.edu](mailto:nhclr@channing.harvard.edu)

**Table of Contents:**

1. **Designing Sex-specific Transcription Factor-Gene Motif Prior**
2. **Designing Protein-protein Interaction Prior**
3. **Sex Difference in anti PD-1 and anti PDL-1 Inhibitors in Non-small Cell Lung Cancer**
4. **Additional Figures**
5. **Additional Tables**
6. **Understanding Gene Regulatory Sex Differences in LUAD in East Asian Population**
7. **Designing Sex-specific Transcription Factor-Gene Motif Prior**

The prior regulatory network is a binary network of transcription factors to their target genes, where the edges (0 or 1) indicate whether a transcription factor motif exists in a target gene’s promoter. To create the prior regulatory network, we downloaded Homo sapiens transcription factor motifs with direct/inferred evidence from the Catalog of Inferred Sequence Binding Preferences CIS-BP Build 2.0 (http://cisbp.ccbr.utoronto.ca). We mapped these transcription factor position weight matrices (PWM) to the human genome (hg38) using FIMO [1] and retained highly significant matches (p<10-5) that occurred within the promoter regions of Ensembl genes (Gencode v39; annotations downloaded from http://genome.ucsc.edu/cgi-bin/hgTables); promoter regions were defined as [-750; +250] base pairs around the transcription start site (TSS). This process resulted in an initial map of potential regulatory interactions involving 997 transcription factors targeting 61,485 genes. To statistically compare networks, the same set of edge combinations need to be included in both sexes, therefore we created sex-informed transcription factor regulatory priors to account for the lack of Y chromosome genes in females. In the female regulatory prior, edges from or to Y chromosome genes were downweighed to zero, which consisted of 52,266 edges.

1. **Designing Protein-protein Interaction Prior**

We used the STRINGdb Bioconductor package [2] to access and download PPI data from the StringDB database (STRING.version 11.5). We filtered the PPI data to keep only those interactions present between transcription factors in the prior network (score threshold index of 0). PPI scores were normalized by dividing them by 1000 to have a uniform range between 0 and 1 for the PPI and the motif prior network. We set transcription factor self-interaction equal to one. Since PPI are undirected, we converted the data into a symmetric form.

1. **Sex Difference in anti PD-1 and anti PDL-1 Inhibitors in Non-small Cell Lung Cancer**

**Table C.1:** Sex difference in response to cancer therapeutics targeting PD-1 and PDL-1. All studies are based on non-small cell lung cancer clinical trials. The last column reports the hazard ratio for overall survival or progression-free survival. The following information is obtained from [3], except the target information is extracted from the corresponding clinical trial.

| **Clinical Trial** | **Target** | **Treatment** | **No. Samples** | | **Hazard Ratio** |
| --- | --- | --- | --- | --- | --- |
|  |  |  | Male | Female |  |
| [4] | PD-1 | Pembrolizumab | 425 | 266 | Male: 0.7 |
|  |  | Docetaxel | 209 | 134 | Female: 1.02 |
| [5] | PD-1 | Pembrolizumab | 92 | 62 | Male: 0.39 |
|  |  | Chemotherapy | 95 | 56 | Female: 0.75 |
| [6] | PDL-1 | Atezolizumab | 261 | 164 | Male: 0.79* |
|  |  | Docetaxel | 259 | 166 | Female: 0.64* |
| [7] | PD-1 | Nivolumb | 184 | 87 | Male: 1.05 |
|  |  | Chemotherapy | 148 | 122 | Female: 1.36 |
| [8] | PDL-1 | Durvalumab | 334 | 142 | Male: 0.54 |
|  |  | Placebo | 166 | 71 | Female: 0.54 |

*Hazard ratio is computed based on overall survival, for all other studies hazard ratio is computed based on progression free survival.

1. **Additional Figures**

**Figure D.1:** Sex difference in LGRC control lung samples within nonsmokers and smokers. Normalized enrichment scores (NES) from GSEA using KEGG pathways are shown for all pathways that have significant (adjusted p-value < 0.05) sex difference among either nonsmokers or smokers in LGRC. A positive NES corresponds to higher targeting in females and a negative NES corresponds to higher targeting in males. Pathways with higher targeting in male are marked blue and pathways with higher targeting in female are marked red. Green boxes highlight pathways associated with cell proliferation and brown boxes highlight pathways associated with environmental carcinogen metabolism.


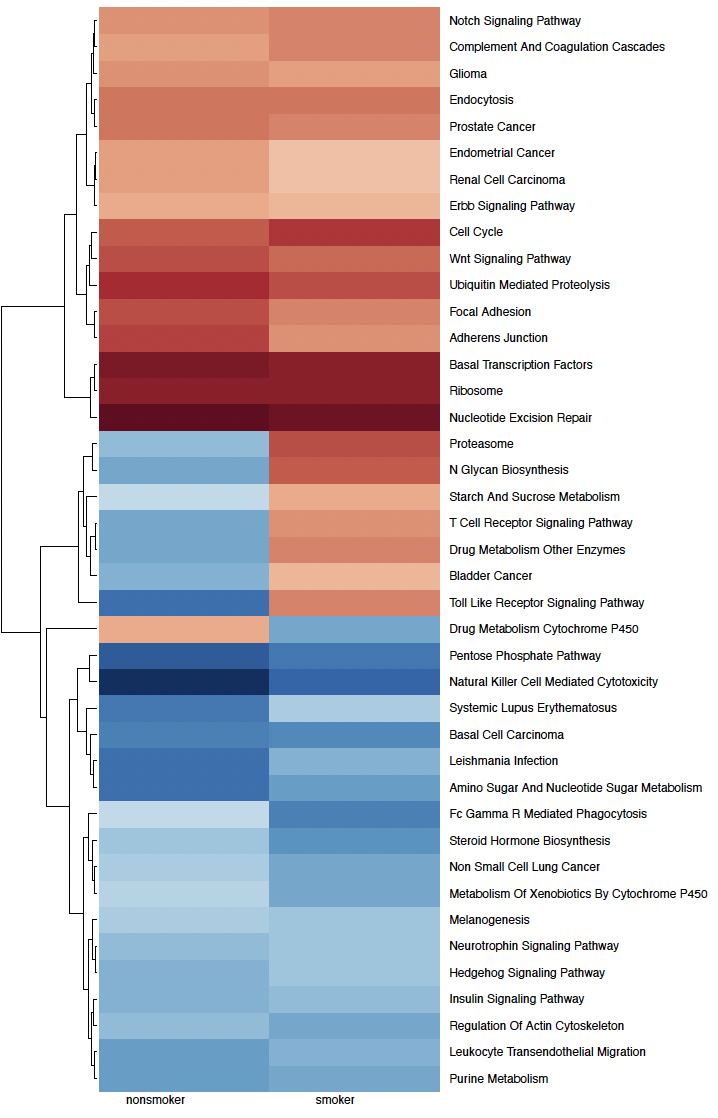

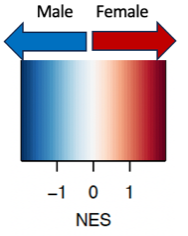


**Figure D.2**: Sex difference in tumor samples from the validation data GSE68465 within nonsmokers and smokers. Normalized enrichment scores (NES) from GSEA using KEGG pathways are shown for all pathways that have significant (adjusted p-value < 0.05) sex difference among either nonsmokers or smokers (in TCGA). A positive NES corresponds to higher targeting in females and a negative NES corresponds to higher targeting in males. Pathways with higher targeting in male are marked blue and pathways with higher targeting in female are marked red. Green boxes highlight pathways associated with cell proliferation and purple boxes highlight pathways associated with immune response.


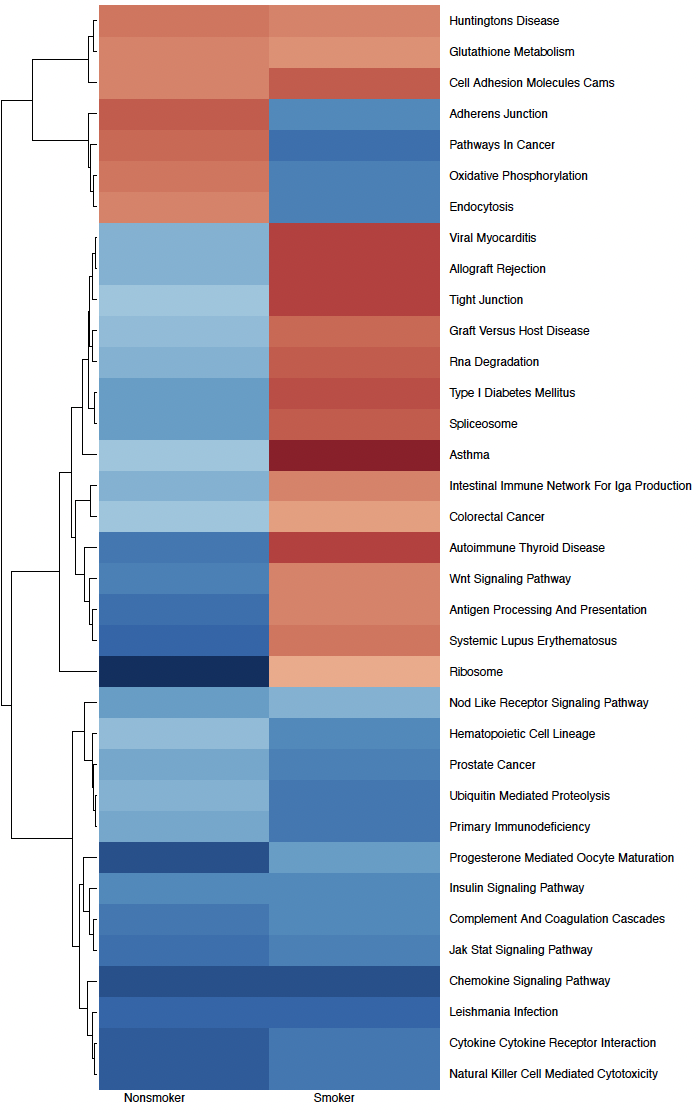

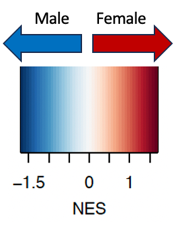


**Figure D.3**: Heatmap representing the t-statistics corresponding to the sex differences in the ribosomal pathway from the limma model for the samples from both GTEx and TCGA, split by their smoking history. From the heatmap we observed that the targeting patterns of the 85 ribosomal genes included in our analysis is distinct among GTEx nonsmokers, compared to the other three groups. A positive value of the t-statistics corresponds to higher targeting in females and a negative value corresponds to higher targeting in males. Genes with higher targeting in males are marked blue and genes with higher targeting in females are marked red.


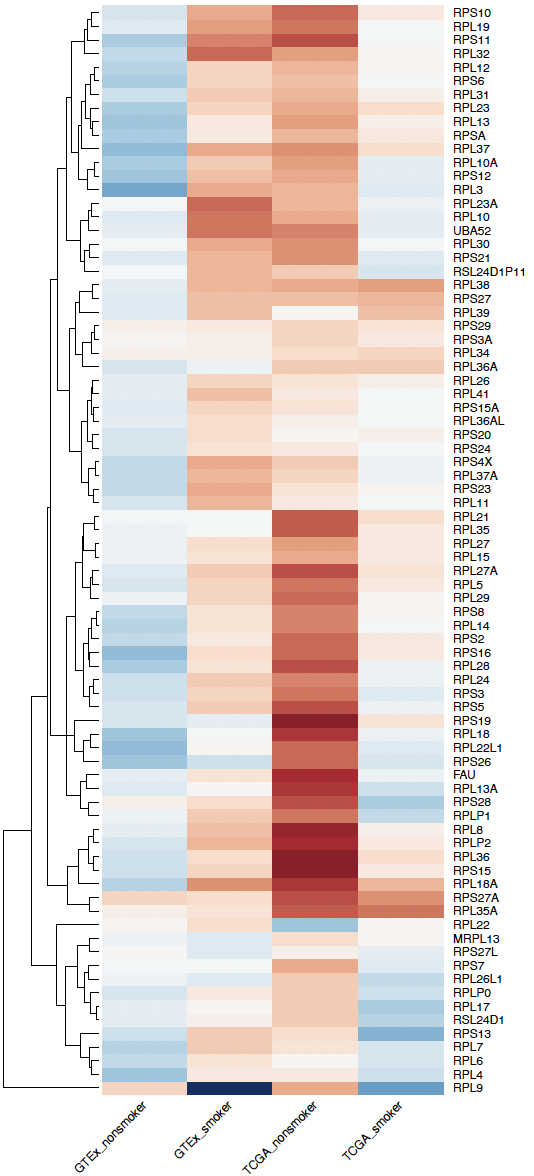

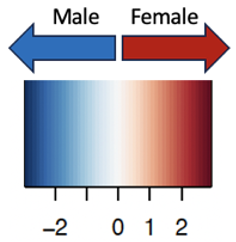


**Figure D.4**: Sex difference in tumor samples from the validation data GIS031 within nonsmokers and smokers. Normalized enrichment scores (NES) from GSEA using KEGG pathways are shown for all pathways that have significant (adjusted p-value < 0.05) sex difference among either nonsmokers or smokers (in TCGA). A positive NES corresponds to higher targeting in females and a negative NES corresponds to higher targeting in males. Pathways with higher targeting in male are marked blue and pathways with higher targeting in female are marked red. Green boxes highlight pathways associated with cell proliferation and purple boxes highlight pathways associated with immune response.

**
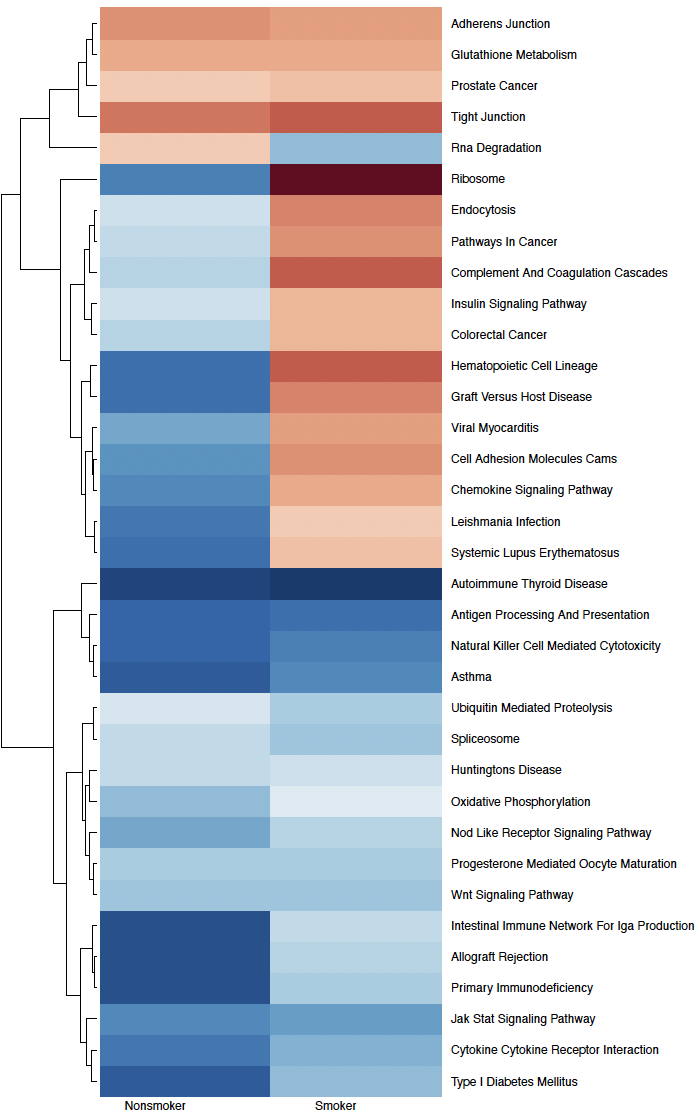
**
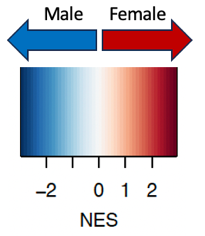


**Figure D.5**: Sex difference in immune and stromal cell composition in GTEx samples: nonsmokers (left) and smokers (right). Cell compositions are computed using “xcell”, which derives cell composition proportion of 36 immune and stromal, along with three composite scores: immune score, stroma score and microenvironment score. The bubbleplot shows only those cells that are significantly (p-value < 0.05) different in proportion in male and female samples.


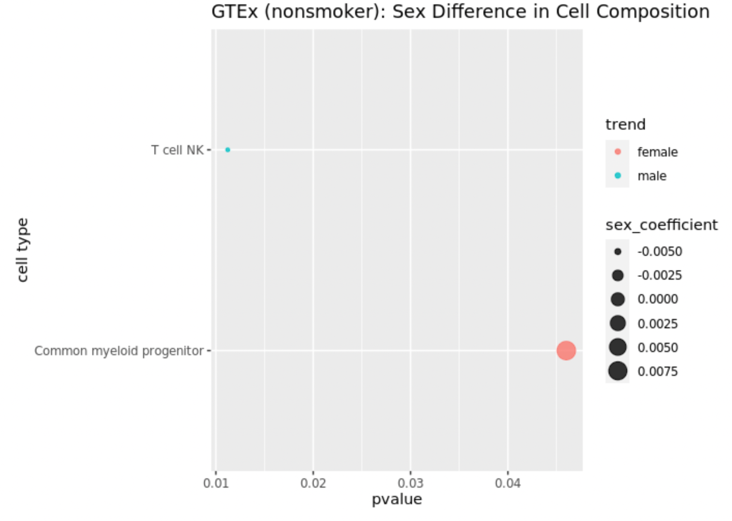

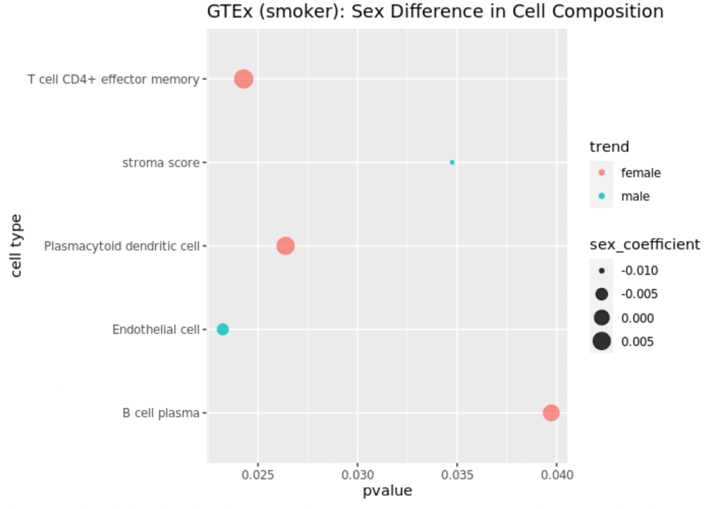


**Figure D.6: Defining biological sex based on sex chromosome complement.** Scatterplot of first two principal components of Y chromosome gene expression in GTEx (top left), TCGA (top right), LGRC (bottom left) and GSE68465 (bottom right).


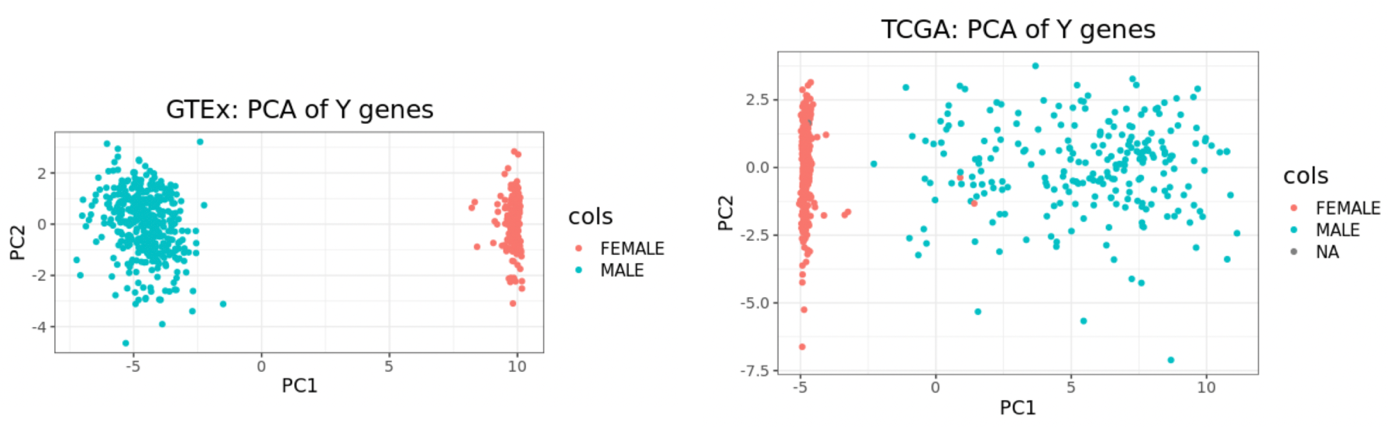


**
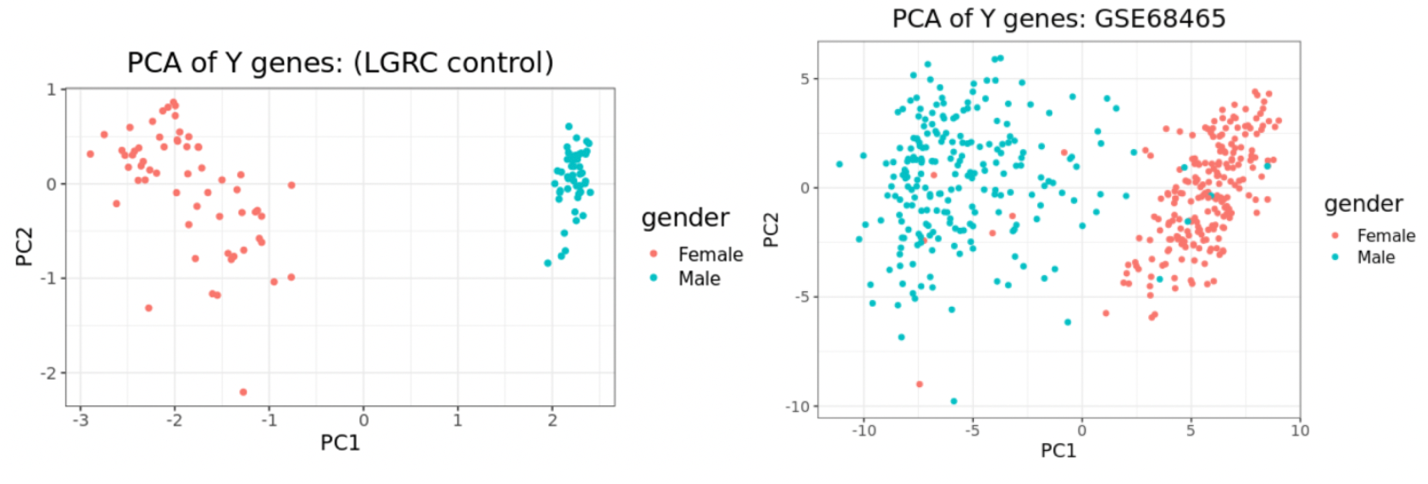
**

**Figure D.7**: Violinplot representing the distribution of the expression of *XIST* gene among samples from GTEx (left) and TCGA (right), split by smoking history.

**
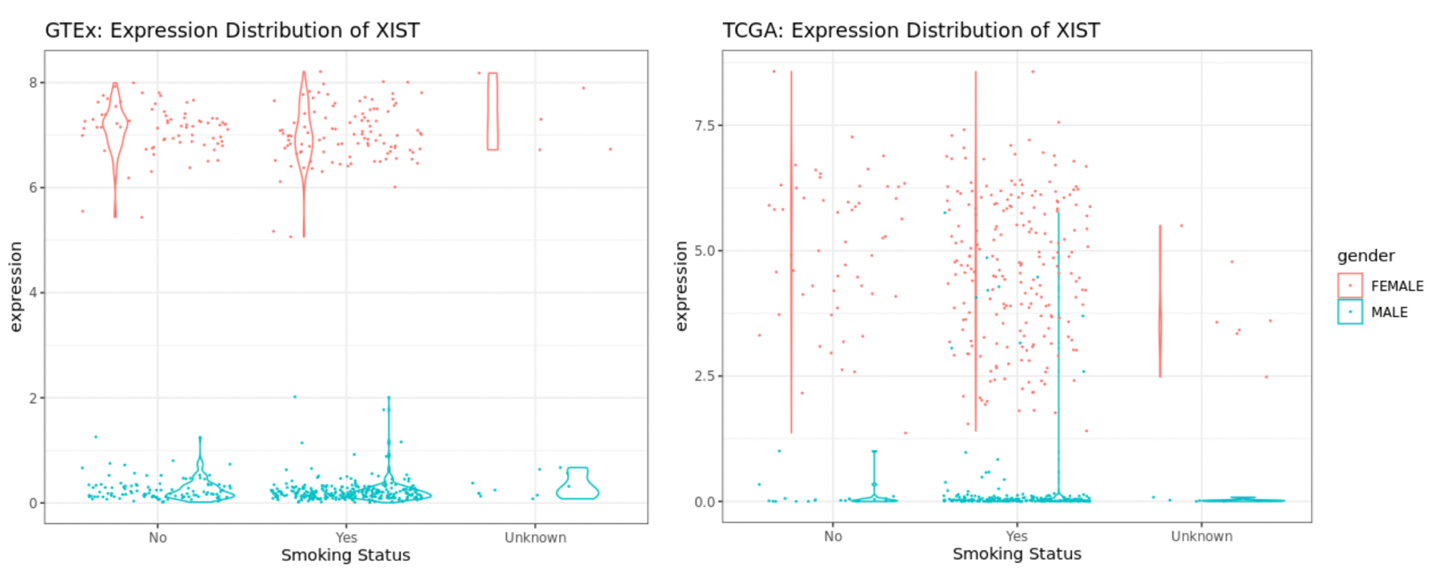
**

1. **Additional Tables**

**Table E.1:** Distribution of Clinical Variables by Sex in GTEx.

**
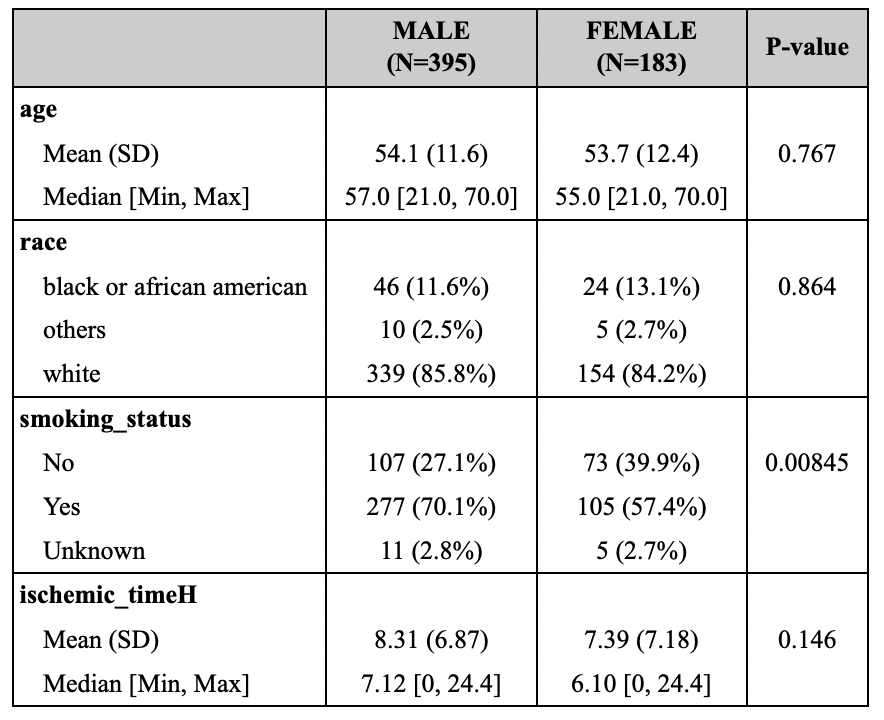
**

**Table E.2:** Distribution of Clinical Variables by Sex in TCGA.

**
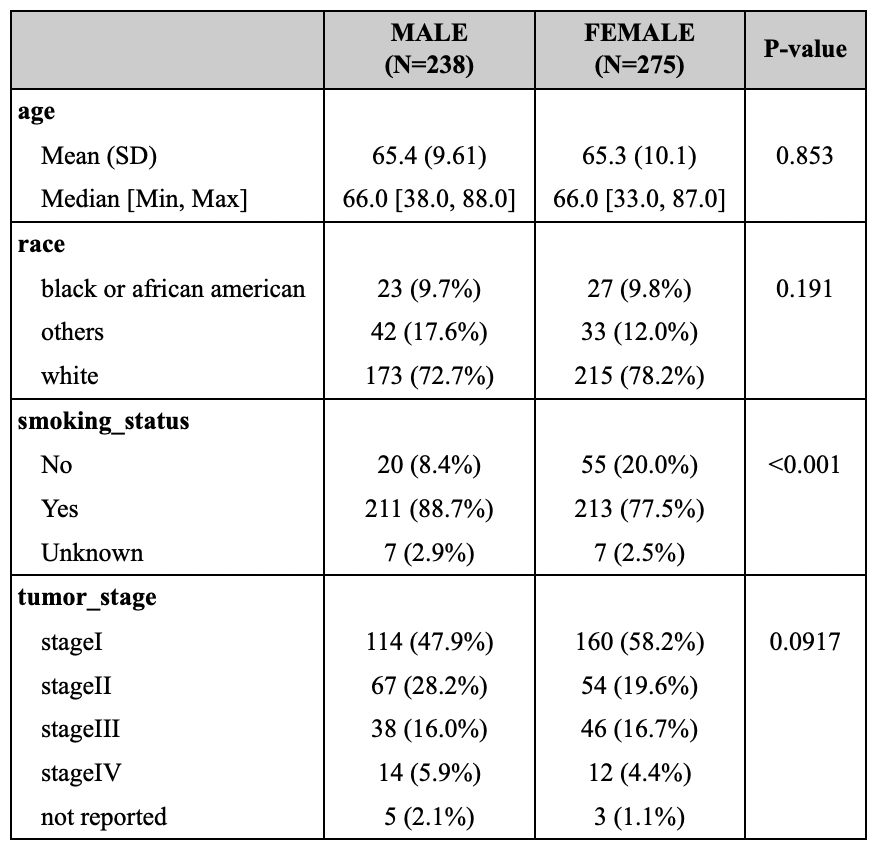
**

**Table E.3:** Distribution of Clinical Variables by Sex in LGRC.

**
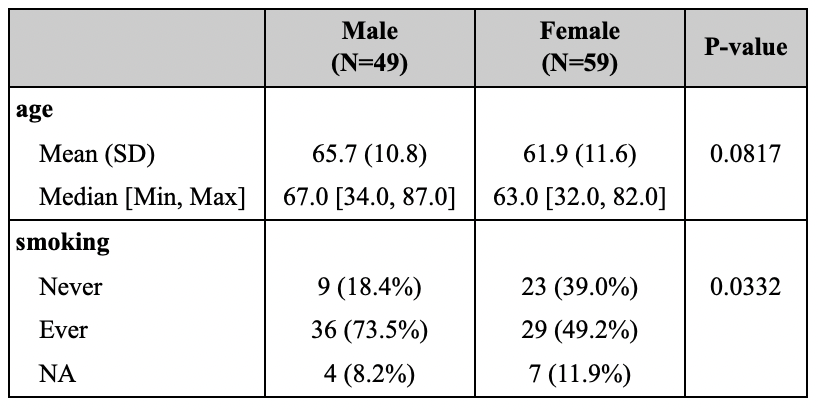
**

**Table E.4:** Distribution of Clinical Variables by Sex in GSE68465.

**
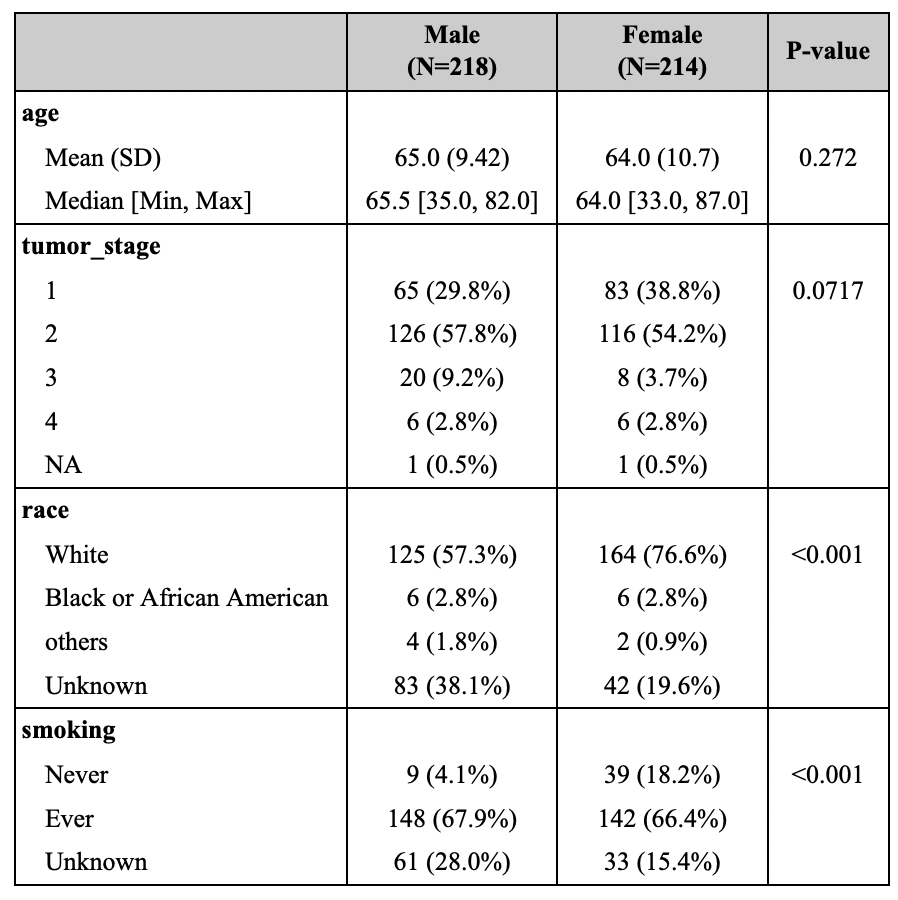
**

**Table E.5:** Distribution of Clinical Variables among individuals who received chemotherapy versus those who did not, in TCGA dataset.


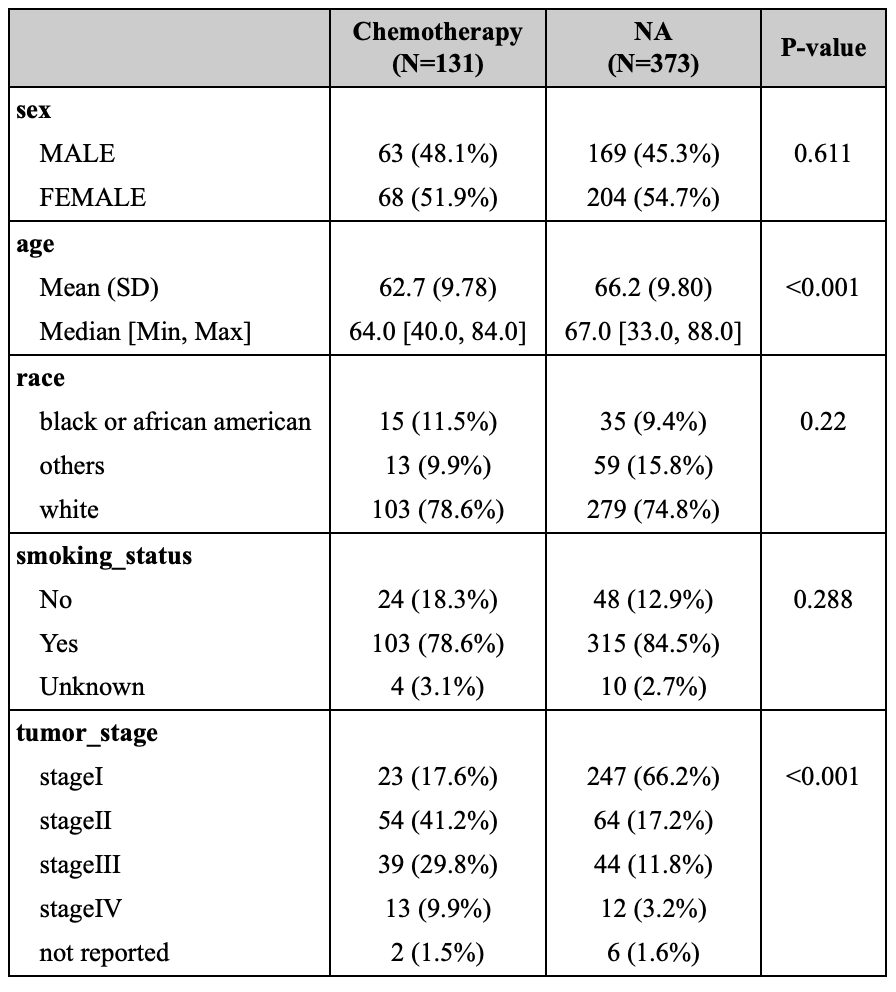


1. **Understanding Gene Regulatory Sex Differences in LUAD in East Asian Population**

In the analysis presented in the paper, the cohorts of the discovery and validation datasets (TCGA and GSE68465 respectively) consisted primarily of White and African American individuals. To test whether our results could be generalized to populations with other racial and ethnic backgrounds, we analyzed an independent dataset consisting of 169 LUAD tumor samples (75 males and 94 females) from only East Asian individuals, downloaded from OncoSG, the Singapore Oncology Data Portal (<https://src.gisapps.org/OncoSG/>; OncoSG accession GIS031). We removed 538 genes from GIS031 that were not expressed in any samples. For genes with multiple transcripts, we computed the mean expression of all transcript representatives for each individual and used that value as the gene’s expression level, leaving a total of 18200 genes for the final analysis; only 16211 of these were also present in TCGA. Since the downloaded data from GIS031 were already RPKM-normalized (instead of the TPM values we used for GTEx and TCGA) and consisted of many genes not reported for the TCGA, we performed a separate analysis on GIS031, following the pipeline used on TCGA, instead of performing a joint analysis combining GIS031 and TCGA.

We observed (**Figure D.4**) that pathways associated with cell adhesion and proliferation including pathways in cancer, cell adhesion molecules (CAMs) and WNT signaling pathway have higher targeting in males than females in nonsmokers, consistent with the results obtained from TCGA data. However, unlike in TCGA for smokers, these same pathways with the exception of WNT signaling pathway, are more highly targeted in females than males.

Immune pathways were also targeted in a sex-biased manner. However, the patterns of sex differences in GIS031 were different from those observed in TCGA. In TCGA, immune pathways including antigen processing and presentation, natural killer cell mediated cytotoxicity, allograft rejection, primary immunodeficiency, and JAK-STAT signaling pathway, all had higher targeting in females compared to males; in GIS031, these pathways had greater targeting in males, irrespective of smoking history.

Based on the patterns of sex differences observed in TCGA (which predominantly consists of White and African American individuals) and in GIS031 (which consists of only East Asian individuals), there may be gene regulatory differences between males and females that depend on race. Indeed, some studies have reported sex differences in gene expression of key biological pathways including several immune pathways are distinct between populations of varying race [9]. However, it is worth noting that some of the disparity in our findings between TCGA and GIS031 might have resulted from a difference in data processing (TPM vs RPKM normalization) and because of the use of different sets of genes.

# Bibliography

| [1] | C. E. Grant, T. L. Bailey and W. S. Noble, "FIMO: scanning for occurrences of a given motif,," *Bioinformatics,* vol. 27, no. 7, 2011. |
| --- | --- |
| [2] | D. Szklarczyk, A. L. Gable, K. C. Nastou and D. Lyon et. al, "The STRING database in 2021: customizable protein-protein networks, and functional characterization of user-uploaded gene/measurement sets," *Nucleic Acids Research (Database issue),* vol. 49, 2021. |
| [3] | A. Grassadonia, I. Sperduti and P. Vici et. al, "Effect of Gender on the Outcome of Patients Receiving Immune Checkpoint Inhibitors for Advanced Cancer: A Systematic Review and Meta-Analysis of Phase III Randomized Clinical Trials," *J Clin Med.,* vol. 7, no. 12, p. 542, 2018 Dec 12. |
| [4] | R. Herbst, P. Baas and D. W. Kim et. al, "Pembrolizumab versus docetaxel for previously treated, PD-L1-positive, advanced non-small-cell lung cancer (KEYNOTE-010): A randomised controlled trial," *Lancet,* vol. 387, p. 1540–1550, 2016. |
| [5] | M. Reck, D. Rodríguez-Abreu and A. Robinson et. al, "Pembrolizumab versus chemotherapy for PD-L1-positive non-small-cell lung cancer," *N. Engl. J. Med.,* vol. 375, pp. 1823-1833, 2016. |
| [6] | A. Rittmeyer, F. Barlesi, D. Waterkamp and K. Park et. al, "Atezolizumab versus docetaxel in patients with previously treated non-small-cell lung cancer (OAK): A phase 3, open-label, multicentre randomised controlled trial," *Lancet,* vol. 389, p. 255–265, 2017. |
| [7] | D. Carbone, M. Reck, L. Paz-Ares, B. Creelan and L. Horn et. al, "First-line nivolumab in stage iv or recurrent non-small-cell lung cancer," *N. Engl. J. Med.,* vol. 376, p. 2415–2426, 2017. |
| [8] | S. Antonia, A. Villegas and D. Daniel et. al, "Durvalumab after chemoradiotherapy in stage III non-small-cell lung cancer," *N. Engl. J.Med.,* vol. 377, p. 1919–1929, 2017. |
| [9] | X. Li, S. Wei, L. Deng and H. Tao et. al, "Sex-biased molecular differences in lung adenocarcinoma are ethnic and smoking specific," *BMC Pulmonary Medicine,* vol. 23, no. 1, p. 99, 2023. |
